# Supplementary material for: AlphaFold2 and RoseTTAFold predict posttranslational modifications. Chromophore formation in GFP-like proteins
Source: PLoS One. 2022 Jun 16;17(6):e0267560. doi: 10.1371/journal.pone.0267560 (PMC9202861; doi:10.1371/journal.pone.0267560)

**Fig S1. Main chain “hydrogen bonding” interactions measured and presented in** [**Data S1**](https://docs.google.com/spreadsheets/d/1_Gn-_7APBATI3aSVSg7jUpHuHE9AQ2Ml/edit#gid=932137884). For an ideal α-helix one expects to find hydrogen bonds between all i to i+1 residues (right). In the standard structures that have a chromophore the interactions shown in green were not formed because residues 65,66 and 67 were post-translationally modified.


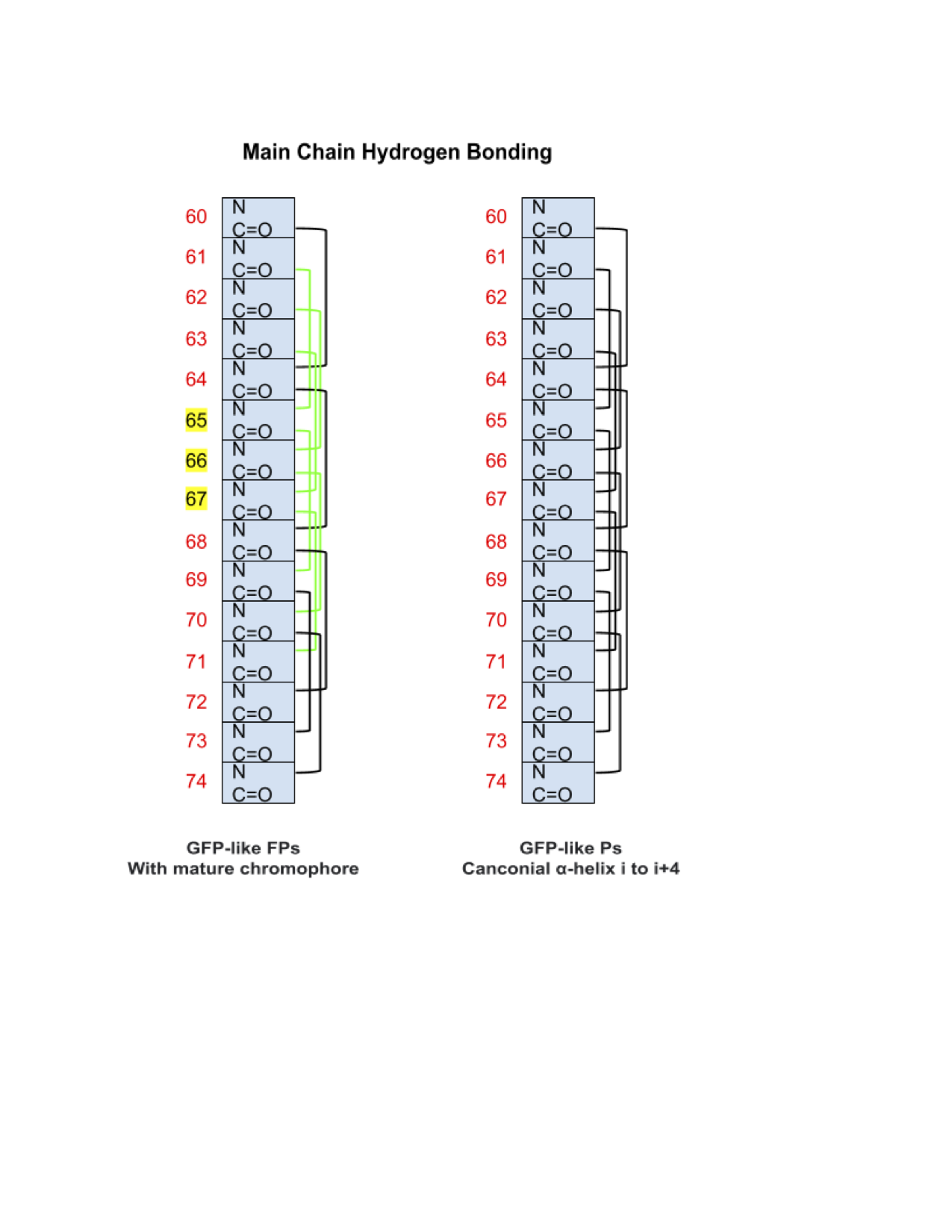

Supplement: S1 Fig — (DOCX) [file pone.0267560.s001.docx]
