# Supplementary material for: AlphaFold2 and RoseTTAFold predict posttranslational modifications. Chromophore formation in GFP-like proteins
Source: PLoS One. 2022 Jun 16;17(6):e0267560. doi: 10.1371/journal.pone.0267560 (PMC9202861; doi:10.1371/journal.pone.0267560)
Supplement: S2 Fig — (DOCX) [file pone.0267560.s002.docx]

**Fig S2.** Alignment of all the sequences used in this paper. Shown relative to S65T GFP**.** Residues highlighted in yellow and blue make up the central helix. The 3 residues highlighted in blue correspond to the chromophore forming 65TYG67 in 1EMA. The origins of the GFP-like proteins are given in Table S1.

**
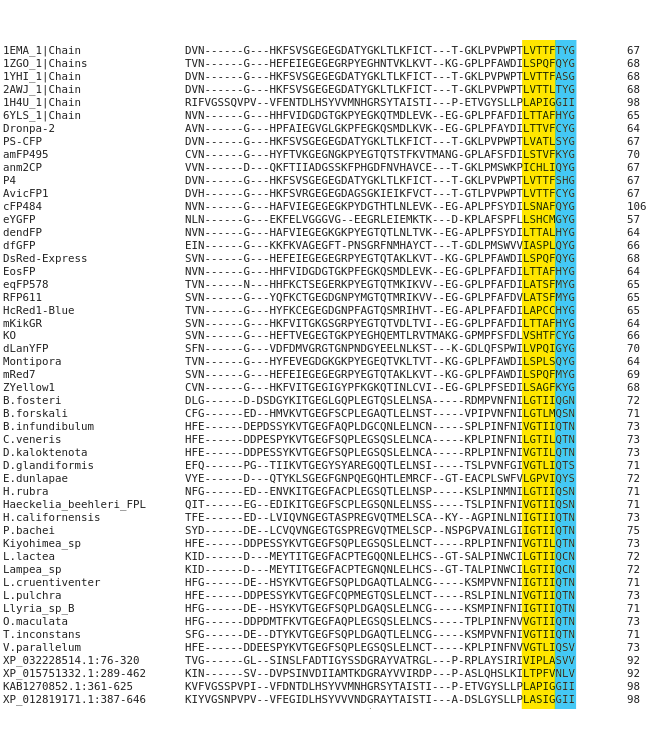
**

**
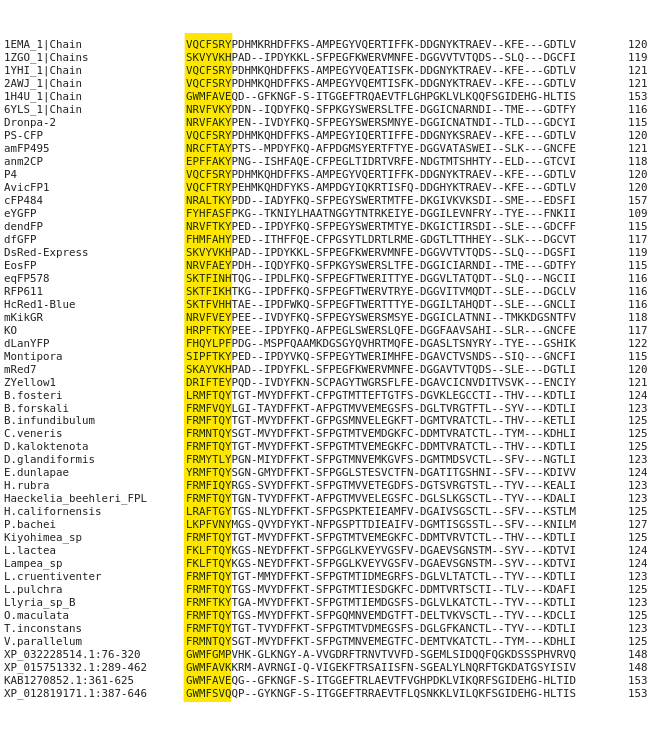
**
