# Supplementary material for: AlphaFold2 and RoseTTAFold predict posttranslational modifications. Chromophore formation in GFP-like proteins
Source: PLoS One. 2022 Jun 16;17(6):e0267560. doi: 10.1371/journal.pone.0267560 (PMC9202861; doi:10.1371/journal.pone.0267560)

**Fig S3.** Prediction results using LASSO model from Table S3 on AlphaFold2 data. GFP-like proteins that will form a chromophore and those that do not.


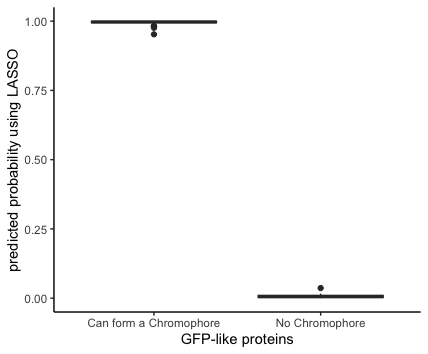

Supplement: S3 Fig — (DOCX) [file pone.0267560.s003.docx]
