# Supplementary material for: AlphaFold2 and RoseTTAFold predict posttranslational modifications. Chromophore formation in GFP-like proteins
Source: PLoS One. 2022 Jun 16;17(6):e0267560. doi: 10.1371/journal.pone.0267560 (PMC9202861; doi:10.1371/journal.pone.0267560)

Fig S4. RMSD overlap of the α-helix of the 1EMA-crystal with the α-helix of 1EMA as determined by RoseTTAFold for GFP-like proteins that will form a chromophore and those that do not.


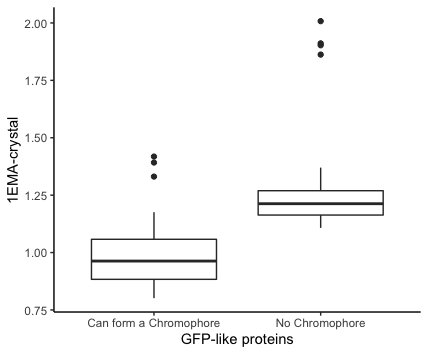

Supplement: S4 Fig — (DOCX) [file pone.0267560.s004.docx]
