# Supplementary material for: AlphaFold2 and RoseTTAFold predict posttranslational modifications. Chromophore formation in GFP-like proteins
Source: PLoS One. 2022 Jun 16;17(6):e0267560. doi: 10.1371/journal.pone.0267560 (PMC9202861; doi:10.1371/journal.pone.0267560)

Fig S5. Predicted possibility that structure forms a chromophore or cannot form a chromophore using LASSO model from Table S5, which is based on RoseTTAFold data.


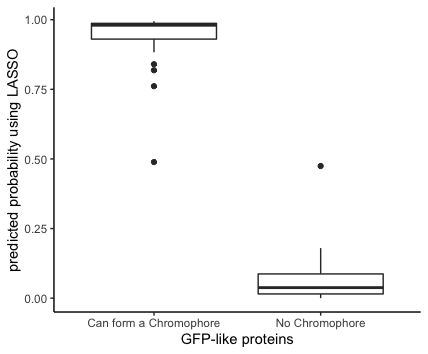

Supplement: S5 Fig — (DOCX) [file pone.0267560.s005.docx]
