# Supplementary material for: AlphaFold2 and RoseTTAFold predict posttranslational modifications. Chromophore formation in GFP-like proteins
Source: PLoS One. 2022 Jun 16;17(6):e0267560. doi: 10.1371/journal.pone.0267560 (PMC9202861; doi:10.1371/journal.pone.0267560)
Supplement: S1 Table — (DOCX) [file pone.0267560.s007.docx]

**Table S1.** Description of all sequences used in this study.

|  | **Name** | **Percent Identity to 1EMA** | **Species** | **Source** |
| --- | --- | --- | --- | --- |
| **Standards** | 1EMA[28] | 100 | *Aequorea victoria* | [rcsb.org](http://www.rcsb.org/) |
|  | 1YHI[40] | 97 | *Aequorea victoria* |  |
|  | 2AWJ[29] | 97 | *Aequorea victoria* |  |
|  | 1ZGO[41] | 23 | *Discosoma* |  |
|  | 6YLS[42] | 25 | *Lobophyllia hemprichii* |  |
|  | 1H4U[30] | 17 | *Mus musculus* |  |
| **Capable of forming**  **chromophore** | Dronpa-2[43] | 24 | *Echinophyllia sp. SC22* | <https://www.fpbase.org/lineage/> |
|  | PS-CFP[44] | 89 | *Aequorea coerulescens* |  |
|  | amFP495[45] | 25 | *Anemonia majano* |  |
|  | anm2CP[46] | 25 | *Anthoathecata* |  |
|  | P4[47] | 98 | *Aequorea victoria* |  |
|  | AvicFP1[48] | 80 | *Aequorea victoria* |  |
|  | cFP484[49] | 26 | *Clavularia sp.* |  |
|  | eYGFP[50] | 17 | *Chiridius poppei* |  |
|  | dendFP[51] | 26 | *Dendronephthya sp.* |  |
|  | dfGFP[52] | 28 | *Olindias formosus* |  |
|  | DsRed-Express[53] | 23 | *Discosoma sp.* |  |
|  | EosFP[54] | 26 | *Lobophyllia hemprichii* |  |
|  | eqFP578[55] | 19 | *Entacmaea quadricolor* |  |
|  | RFP611[56] | 21 | *Entacmaea quadricolor* |  |
|  | HcRed1-Blue[57] | 20 | *Heteractis crispa* |  |
|  | mKikGR[58] | 27 | *Favia favus* |  |
|  | KO[59] | 23 | *Verrillofungia concinna* |  |
|  | dLanYFP[60] | 24 | *Branchiostoma lanceolatum* |  |
|  | *Montipora sp.* #20[61] | 23 | *Montipora sp.* #20 |  |
|  | mRed7[62] | 30 | synthetic |  |
|  | zYellow1 | 22 | *Zoanthus sp.* |  |
| **Incapable of forming chromophore** | *B. forskali[32]* | 20 | *B. forskali[32]* | Non-excitable fluorescent protein orthologs found in ctenophores [32] |
|  | *B. fosteri[32]* | 20 | *B. fosteri[32]* |  |
|  | *B. infundibulum[32]* | 18 | *B. infundibulum[32]* |  |
|  | *C. veneris[32]* | 20 | *C. veneris[32]* |  |
|  | *D. glandiformis[32]* | 23 | *D. glandiformis[32]* |  |
|  | *D. kaloktenota[32]* | 20 | *D. kaloktenota[32]* |  |
|  | *E. dunlapae[32]* | 22 | *E. dunlapae[32]* |  |
|  | *H. californensis[32]* | 18 | *H. californensis[32]* |  |
|  | *H. rubra[32]* | 19 | *H. rubra[32]* |  |
|  | *H. beehleri[32]* | 19 | *H. beehleri[32]* |  |
|  | *Kiyohimea[32]* | 19 | *Kiyohimea[32]* |  |
|  | *L. cruentiventer[32]* | 21 | *L. cruentiventer[32]* |  |
|  | *L. lactea[32]* | 20 | *L. lactea[32]* |  |
|  | *L. pulchra[32]* | 18 | *L. pulchra[32]* |  |
|  | *Lampea[32]* | 20 | *Lampea[32]* |  |
|  | *Llyria[32]* | 21 | *Llyria[32]* |  |
|  | *O. maculata[32]* | 19 | *O. maculata[32]* |  |
|  | *P. bachei[32]* | 17 | *P. bachei[32]* |  |
|  | *T. inconstans[32]* | 20 | *T. inconstans[32]* |  |
|  | *V. parallelum[32]* | 18 | *V. parallelum[32]* |  |
|  | KAB1270852 | 18 | *Camelus dromedarius* | <https://blast.ncbi.nlm.nih.gov/Blast.cgi?PAGE=Proteins> |
|  | XP012819171 | 17 | *Xenopus tropicalis* |  |
|  | XP032228514 | 12 | *Nematostella vectensis* |  |
|  |  |  |  |  |
