# Supplementary material for: AlphaFold2 and RoseTTAFold predict posttranslational modifications. Chromophore formation in GFP-like proteins
Source: PLoS One. 2022 Jun 16;17(6):e0267560. doi: 10.1371/journal.pone.0267560 (PMC9202861; doi:10.1371/journal.pone.0267560)
Supplement: S2 Table — (DOCX) [file pone.0267560.s008.docx]

**Table S2.** Summary statistics of the RMSD overlap (in Angstrom) of the α-helix of the 1EMA-crystal with the α-helix of 1EMA as determined by AlphaFold2 for GFP-like proteins that will form a chromophore and those that do not.

| Groups | Minimum | First Quartile | Median | Mean | Third Quartile | Maximum |
| --- | --- | --- | --- | --- | --- | --- |
| Does not form  chromophore | 1.165 | 1.244 | 1.274 | 1.372 | 1.320 | 2.086 |
| Forms  chromophore | 0.574 | 0.734 | 0.784 | 0.800 | 0.860 | 1.049 |
