# Supplementary material for: AlphaFold2 and RoseTTAFold predict posttranslational modifications. Chromophore formation in GFP-like proteins
Source: PLoS One. 2022 Jun 16;17(6):e0267560. doi: 10.1371/journal.pone.0267560 (PMC9202861; doi:10.1371/journal.pone.0267560)
Supplement: S3 Table — (DOCX) [file pone.0267560.s009.docx]

**Table S3.** LASSO model results for the alpha helix overlap with 1EMA-crystal as well as H-bond distances in Angstrom between residues 61-65 (HD2), 62-66 (HD3), 70-74 (HD11) collected using AlphaFold2.

| Variable names | coefficients | exp(coefficients) |
| --- | --- | --- |
| 1EMA-crystal | -7.284 | 0.001 |
| HD2 | 0.395 | 1.485 |
| HD3 | 0.745 | 2.107 |
| HD11 | 0.788 | 2.199 |

From the model results (as listed above in Table S3) The first column listed the variables that are selected out, the second column are the coefficients estimates, and the third column are the exponentiated coefficients. Each exponentiated coefficient is the change in odds in the multiplicative scale for a unit increase in the corresponding predictor variable holding other variables at certain value. For example, the exponentiated coefficient for 1EMA-crystal is 0.001, meaning that holding the other variables at a fixed value, with one unit increase in the alpha helix overlap of 1EMA-crystal, the odds of being GFP-like proteins that will form a chromophore will decrease by 99.9% (=100%-0.1%). Also, from the boxplot of the predictions of the LASSO model against the two groups, we can see there is a clear distinction.
