# Supplementary material for: AlphaFold2 and RoseTTAFold predict posttranslational modifications. Chromophore formation in GFP-like proteins
Source: PLoS One. 2022 Jun 16;17(6):e0267560. doi: 10.1371/journal.pone.0267560 (PMC9202861; doi:10.1371/journal.pone.0267560)
Supplement: S4 Table — (DOCX) [file pone.0267560.s010.docx]

**Table S4.** Summary statistics of the RMSD overlap (in Angstrom) of the α-helix of the 1EMA-crystal with the α-helix of 1EMA as determined by RoseTTAFold for GFP-like proteins that will form a chromophore and those that do not.

| Groups | Minimum | First Quartile | Median | Mean | Third Quartile | Maximum |
| --- | --- | --- | --- | --- | --- | --- |
| Do Not Form  Chromophore | 1.1074 | 1.16335 | 1.21275 | 1.317071 | 1.269375 | 2.0078 |
| Will Form  Chromophore | 0.8014 | 0.8834 | 0.963 | 1.003562 | 1.057825 | 1.4181 |
