# Supplementary material for: AlphaFold2 and RoseTTAFold predict posttranslational modifications. Chromophore formation in GFP-like proteins
Source: PLoS One. 2022 Jun 16;17(6):e0267560. doi: 10.1371/journal.pone.0267560 (PMC9202861; doi:10.1371/journal.pone.0267560)
Supplement: Attachment — Submitted filename: Response to Reviewers.doc [file pone.0267560.s012.doc]

April 5, 2022

Dear Dr. Maria Gasset,

Thank you for facilitating the peer review of our manuscript. Sorry you had so much difficulty in finding reviewers. We were pleased to see that the reviewer was so positive about the manuscript and only had two minor suggestions. Here are our responses to the suggestions.

1) Maybe a figure with a representative structure of a GFP-like protein with fluorophore investigated in this study would be useful, as well as such a protein without a fluorophore.

I don’t think a figure such as the one suggested above will contribute much to the understanding of the work presented. They are essentially the same because the deep learning programs show the conformation prior to post-translational cyclization and do not show the mature chromophore. However, we would be happy to insert a figure like the one shown below.

Fig xx. AlphaFold prediction of the folding of a GFP-like protein with chromophore (left) and without (right) a chromophore. Shown here are the predicted structures of dronpa-2 (chromophore) and the camel nidogen KAB1270852.1 (no-chromophore). They are essentially the same because the deep learning programs show the conformation prior to post-translational cyclization and do not show the mature chromophore.

2) By chromophore, the authors mean a structure that fluoresces, not one that just absorbs light, is that correct?

Yes, we have clarified this by adding the following definition at the first occurrence of the word chromophore. “chromophore (the part of the protein responsible for fluorescence)”.

We have also made numerous stylistic changes to manuscript so that it corresponds to the PLOS One format. These changes have not been monitored with the track changes function.

Yours Sincerely,

Marc Zimmer

Jean Tempel ’65 Professor of Chemistry
